# The longitudinal relationship between depression and anxiety in colorectal cancer patients undergoing chemotherapy and family caregivers: A cross lagged panel model

**DOI:** 10.1371/journal.pone.0319622

**Published:** 2025-04-16

**Authors:** Qingfeng Wang, Lijuan Zeng, Li Gao, Huiqiong Xu

**Affiliations:** 1 Department of Abdominal Oncology, West China Hospital, Chengdu, Sichuan, China; 2 Division of Abdominal Tumor Multimodality Treatment, Cancer Center, West China Hospital, Sichuan University/West China School of Nursing, Chengdu, Sichuan, China; The Chinese University of Hong Kong, HONG KONG

## Abstract

**Background:**

Family caregivers play a crucial caregiving role for colorectal cancer patients undergoing chemotherapy, and the emotional states of both patients and caregivers can influence each other. A high prevalence of depression and anxiety exists among both patients and caregivers, with their emotional states mutually influencing each other. This significantly impacts the quality of life for both parties. However, there is limited research on the bidirectional relationship between depression and anxiety in both groups.

**Objective:**

This study aims to investigate the longitudinal bidirectional relationship between depression and anxiety in colorectal cancer patients and their family caregivers using a cross-lagged panel model.

**Method:**

A total of 244 pairs of colorectal cancer patients undergoing chemotherapy and their family caregivers were assessed using the Hospital Anxiety and Depression Scale. Data collection was conducted at four time points: the initial chemotherapy session and 1-, 3-, and 6- months post-chemotherapy. A cross-lagged panel model was employed to analyze the longitudinal interrelationship between depression and anxiety within and between the two groups.

**Result:**

The study found high prevalence rates of depression and anxiety in both colorectal cancer patients and their caregivers. The cross-lagged model revealed a dynamic, bidirectional relationship between depression and anxiety in patients and caregivers from the second wave onwards (*P* <  0.05).

**Conclusion:**

The emotional states of depression and anxiety in colorectal cancer patients and their caregivers show dynamic changes and are longitudinally interrelated. These findings underscore the importance of early psychological assessment and interventions targeting both patients and caregivers.

## Introduction

Colorectal cancer is one of the most prevalent and malignant tumors of the digestive system. According to the World Cancer Research Fund International, colorectal cancer remains among the top three cancers globally. Recent statistics indicate that in 2022, approximately 1,926,425 new cases of colorectal cancer were diagnosed worldwide, resulting in about 1,465,258 deaths from the disease [1]. In China, the incidence of colorectal cancer has been rising rapidly, surpassing growth rates observed in European and North American countries [2]. According to the 2022 national cancer statistics from the National Cancer Institute of China, there were 517,100 new cases of colorectal cancer and 240,000 deaths, positioning colorectal cancer as the second most common cancer in terms of incidence and the fourth in terms of mortality [3]. This increasing burden has significant implications for public health, the economy, and healthcare systems, both in China and globally.

Colorectal cancer patients often experience a high psychological burden due to the disease’s severity and the complexity of its treatments, particularly chemotherapy. Anxiety and depression are highly prevalent in this population, with prevalence rates ranging from 1.0% to 47.2% for anxiety and 1.6% to 57% for depressive symptoms [4,5]. Chemotherapy, though a primary treatment modality, can exacerbate these psychological burdens due to its cyclical nature and adverse effects. Research has shown that chemotherapy acts as a risk factor for inducing anxiety and depression in patients with digestive tract tumors, including colorectal cancer [6]. These psychological symptoms can significantly reduce the quality of life of patients undergoing chemotherapy [7]. Thus, addressing anxiety and depression in colorectal cancer patients during chemotherapy is crucial.

Family caregivers play an integral role in supporting colorectal cancer patients undergoing chemotherapy by providing emotional, economic, and daily life support [8]. However, the extended duration of chemotherapy can create tension between caregiving responsibilities and the demands of daily life, leading to increased caregiver burden and the onset of negative emotions such as anxiety and depression [9]. Studies show that up to 46.3% of family caregivers of cancer patients experience severe depression, and 53% experience severe anxiety [10]. These emotional burdens not only affect the caregivers’ well-being but also influence the quality of care they provide to the patients, thereby further compromising the patient’s quality of life [11].

The McMaster Family Function Model proposes that families operate as a system where each member influences others, suggesting that a well-functioning family system can alleviate emotional stress and enhance psychological adjustment for both patients and caregivers [12]. Additionally, research highlights the mutual influence of depressive and anxious emotions between cancer patients and their family caregivers, necessitating concurrent psychological support for both parties [13]. Therefore, this study hypothesizes that the depressive and anxious emotions of colorectal cancer patients undergoing chemotherapy and their family caregivers mutually influence one another.

Despite the growing recognition of the psychological burdens experienced by colorectal cancer patients and their caregivers, research on the bidirectional relationship between these emotions during chemotherapy is limited. The objectives of this study are: (1) to assess the prevalence of depressive and anxious emotions in both colorectal cancer patients and their family caregivers at various stages of chemotherapy, (2) to explore the temporal and mutual relationships between these emotional states using a cross-lagged model, and (3) to enhance the understanding of the mechanisms underlying psychological health in this population. By achieving these objectives, this study aims to inform targeted interventions to improve the psychological well-being of both patients and caregivers and to ultimately enhance the quality of care provided.

## Methods

### Research design

This study employs a longitudinal research design. Baseline data were collected through convenience sampling from colorectal cancer patients undergoing inpatient chemotherapy treatment between January 2022 and August 2023, along with their family caregivers. Subsequent follow-up assessments were conducted at the patient’s first chemotherapy session (T1), one month after chemotherapy initiation (T2), three months after chemotherapy initiation (T3), and six months after chemotherapy initiation (T4). The follow-up process involved direct, face-to-face communication with participants to ensure data collection and address any changes in their status.

### Participants

The subjects of this survey were all from the inpatient department of a public hospital in Sichuan Province, the largest comprehensive hospital in southwest China. Trained nursing staff screened inpatient participants and their family caregivers who met the inclusion criteria and invited them to participate in the study under the condition of informed consent.

Inclusion criteria for colorectal cancer patients: (1) Pathologically diagnosed with primary colorectal cancer; (2) Age ≥  18 years; (3) Receiving chemotherapy treatment; and (4) Voluntarily participating with informed consent.

Exclusion criteria for colorectal cancer patients: (1) History of mental illness, personality disorders, visual or hearing impairments; (2) Concurrent other malignant tumors; (3) Concealing the condition; (4) Expected survival time <  6 months; and (5) Death due to various reasons or failure to obtain follow-up data twice consecutively.

Inclusion criteria for family caregivers: (1) Understanding the patient’s condition, being the longest-serving and most burdened caregiver in the family, or being designated as the primary caregiver by the patient; (2) Age ≥  18 years; and (3) Able to read text or answer questions correctly, with informed consent and voluntary participation in the study.

Exclusion criteria for colorectal cancer patients: (1) Requiring compensation; (2) Caregivers who are replaced during the treatment process; and (3) Currently participating in similar studies.

The sample size required for the structural equation model was calculated to be at least 200 participants, based on the minimum sample size guidelines for this statistical approach [14]. Considering a 10% dropout rate, this results in a required sample size of at least 220 cases. In this study, 252 colorectal cancer patients and family caregivers were recruited at baseline. Five cases were lost to follow-up due to patients changing hospitals for chemotherapy treatment, and three cases were excluded due to caregiver replacement during the study. Ultimately, follow-up was completed for 244 patient-caregiver pairs.

### Procedure

The four follow-up visits for patients and their family caregivers were conducted in person, with paper-based questionnaires used for data collection. Before the study began, researchers or trained investigators provided participants with standardized instructions regarding the purpose of the survey and guidance on completing the questionnaires, and informed consent was obtained. The first survey was administered the day before discharge, taking into account the patient’s treatment-related adverse reactions and medical condition. Subsequent surveys were completed after each chemotherapy session. Researchers collected the completed questionnaires on-site, and for participants unable to complete the questionnaire independently, research personnel provided assistance following standardized instructions.

## Measures

### Sociodemographic information

To the research objectives, relevant literature was reviewed and combined with clinical experience to develop a general information survey form. This form includes patient demographic and disease-related data, such as gender, age, educational level, marital status, working status, presence of stoma, and colorectal cancer staging. Additionally, demographic data for caregivers are collected, including gender, age, educational level, marital status, working status, relationship with the patient. The data were obtained through structured interviews and medical records review.

### Chinese version of the Hospital Anxiety and Depression Scale

The Hospital Anxiety and Depression Scale (HADS) is primarily used for screening non-psychiatric anxiety and depression symptoms in hospital settings. It consists of 14 items divided into two subscales: anxiety (7 items) and depression (7 items). Each item is scored on a four-point scale (0-3), with a total subscale score of ≥ 9 indicating the presence of anxiety or depression symptoms. Higher scores indicate more severe symptoms. The scale has been previously applied in research investigating Chinese colorectal cancer patients and family caregivers [15]. In this study, the anxiety subscale Cronbach’s alpha coefficient was 0.87, and the depression subscale Cronbach’s alpha coefficient was 0.91. The assessment method was consistent across all groups in this study.

### Statistical analysis

This study employed SPSS 26.0 for data entry, statistical description, and repeated measures analysis of variance 3. Normally distributed continuous data were presented using mean ±  standard deviation, while non-normally distributed data were expressed as median (P25, P75). Frequency and percentage were used for categorical data.

Repeated measures ANOVA was utilized to compare longitudinal trends in the study. If the anxiety and depression scores of patients and family caregivers followed a normal distribution, Pearson correlation analysis was employed to explore their correlation. Otherwise, Spearman correlation analysis was used.

AMOS 24.0 software was employed to establish a cross-lagged path analysis model to investigate the mutual predictive relationship between patients and their caregivers’ negative emotions. Model fit was assessed using criteria including *χ*^*2*^/df <  5, goodness of fit index (GFI) >  0.9, comparative fit index (CFI) >  0.9, and RMSEA <  0.08, with a significance level set at α =  0.05 for all two-tailed probabilities.

### Ethical considerations

This study received ethical approval from the Ethics Committee of West China Hospital, Sichuan University [No. (1516) in 2021]. Written informed consent was obtained from all participants prior to their involvement in completing the questionnaires, ensuring their understanding of the study’s purpose and procedures. The study adhered to the principles outlined in the Declaration of Helsinki.

## Results

### Descriptive statistics

In this study, the majority of colorectal cancer patients undergoing chemotherapy were male (n =  143, 58.61%), with most aged between 31 and 60 years (n =  131, 53.69%). Among the patients, 178 (72.95%) had an education level below high school, and 193 (79.10%) were either married or cohabiting with a partner. Considerable patients were still employed (n =  147, 60.25%), cancer stage is stage III and above (n =  231, 94.67%), and 211 (86.48%) not had stoma. Regarding family caregivers, a greater proportion were female (n =  152, 62.30%), and 154 (63.11%) caregivers were aged between 31 and 60. More caregivers have a high school education and above (134, 54.92%). Most caregivers were married or cohabiting with a partner (n =  216, 88.52%), and 205 (84.02%) were employed. The primary family caregivers were the patients’ spouses (n =  125, 51.23%) and children (n =  96, 39.34%). See Table 1 for more detailed information.

**Table 1:** Sociodemographic information for Patients and Family Caregivers (n =  244).

Variable	Characteristics of patients (n/%)	Characteristics of family caregivers (n/%)
**Gender**		
Male	143/58.61	92/37.70
Female	101/41.39	152/62.30
**Age**		
18-30	30/12.30	56/22.95
31-60	131/53.69	154/63.11
≥60	83/34.02	34/13.93
**Educational level**		
Less than high school education	178/72.95	110/45.08
High school education and above	66/27.05	134/54.92
**Marital status**		
Married or living together	193/79.10	216/88.52
Widowed	24/9.84	7/2.87
Separated, divorced, and never married	27/11.07	21/8.61
**Working status**		
Working	147/60.25	205/84.02
Not working	97/39.75	39/15.98
**Have stoma**		
Yes	33/13.52	
No	211/86.48	
**Stage of cancer**		
<III	13/5.32	
≥III	231/94.67	
**Relationship with patients**		
Spouse		125/51.23
Parents		12/4.92
Children		96/39.34
Brothers and Sisters		11/4.51

The prevalence of anxiety among colorectal cancer patients undergoing chemotherapy at four-time points was T1 (39.53%), T2 (56.40%), T3 (56.98%), and T4 (52.32%). The prevalence of depression was T1 (36.05%), T2 (61.05%), T3 (66.86%), and T4 (61.63%). Repeated measures ANOVA indicated that there were statistically significant differences in anxiety (*F* =  5.38, *P* < .001) and depression (*F* =  15.15, *P* < .001) scores across these time points. Anxiety scores showed an increasing trend from T1 to T3 and began to decrease at T4, whereas depression scores exhibited a continuous upward trend from T1 to T4 (see Table 2).

**Table 2:** Anxiety and depression scores of patients and family caregivers at four time points (mean ±  SD).

Variable	Anxiety				*F*	Depression				*F*
	T1	T2	T3	T4		T1	T2	T3	T4	
Patients	7.13 ± 0.36	8.35 ± 0.33	8.92 ± 0.34	8.89 ± 0.36	5.38^***^	6.47 ± 0.36	8.74 ± 0.34	9.05 ± 0.30	9.39 ± 0.35	15.15^***^
Caregivers	7.19 ± 0.30	8.13 ± 0.29	8.45 ± 0.31	8.28 ± 0.31	6.30^***^	6.41 ± 0.32	9.44 ± 0.27	9.67 ± 0.30	9.37 ± 0.29	21.54^***^

Note:

*** , *P* <  0.001.

The prevalence of anxiety among caregivers at four time points was T1 (43.60%), T2 (48.84%), T3 (52.33%), and T4 (52.91%). The prevalence of depression was T1 (43.02%), T2 (72.09%), T3 (71.51%), and T4 (66.28%). Repeated measures ANOVA indicated that there were statistically significant differences in anxiety (*F* =  6.30, *P* < .001) and depression (*F* =  21.54, *P* < .001) scores across these time points. Both anxiety and depression scores among caregivers showed an increasing trend from T1 to T3, with a decrease observed at T4 (see Table 2).

### Correlation analysis

Pearson correlation analysis showed that anxiety and depression in patients and caregivers were positively correlated at all four time points (*r* >  0, *P* <  0.001). This satisfies the conditions of correlation and synchrony, consistent with the assumptions of a cross-lagged design (see Table 3).

**Table 3:** Bivariate correlations, of study variables.

Variables	The anxiety of patients				The anxiety of caregivers				The depression of patients				The depression of caregivers			
	T1	T2	T3	T4	T1	T2	T3	T4	T1	T2	T3	T4	T1	T2	T3	T4
**The anxiety of patients**																
T1	1															
T2	0.301^***^	1														
T3	0.096^***^	0.552^***^	1													
T4	0.142^***^	0.304^**^	0.504^***^	1												
**The anxiety of caregivers**																
T1	0.303^***^	0.151^***^	0.117^***^	0.151^***^	1											
T2	0.008^***^	0.417^***^	0.301^***^	0.229^***^	0.242^***^	1										
T3	0.057^***^	0.396^***^	0.461^***^	0.318^***^	0.108^***^	0.512^***^	1									
T4	0.001^***^	0.270^***^	0.315^***^	0.400^***^	0.173^***^	0.473^***^	0.505^**^	1								
**The depression of patients**																
T1	0.890^***^	0.291^***^	0.118^***^	0.108^**^	0.261^***^	0.006*	0.018^***^	0.011^***^	1							
T2	0.333^***^	0.805^***^	0.494^***^	0.344^***^	0.147^***^	0.258^***^	0.305^**^	0.251^***^	0.345^***^	1						
T3	0.129^***^	0.586^***^	0.763^***^	0.461^***^	0.180^***^	0.340^***^	0.504^***^	0.316^***^	0.137^***^	0.545^***^	1					
T4	0.189^***^	0.463^***^	0.565^***^	0.779^***^	0.111^***^	0.244^***^	0.375^***^	0.379^***^	0.200^***^	0.464^***^	0.506^***^	1				
**The depression of caregivers**																
T1	0.391^***^	0.169^***^	0.100^**^	0.042^**^	0.758^***^	0.191^*^	0.039^**^	0.114^***^	0.411^***^	0.167^***^	0.087^***^	0.098^***^	1			
T2	0.061^***^	0.450^***^	0.324^***^	0.306^***^	0.256	0.610^***^	0.490^***^	0.373^***^	0.069^***^	0.406^***^	0.362^***^	0.187^***^	0.192^***^	1		
T3	0.020^*^	0.272^***^	0.375^***^	0.279^***^	0.189^***^	0.428^***^	0.544^***^	0.373	0.033^***^	0.281^***^	0.366^***^	0.288^***^	0.110^***^	0.462^***^	1	
T4	0.084^***^	0.297^***^	0.330^***^	0.461^***^	0.244^***^	0.374^***^	0.419^***^	0.609^***^	0.085^***^	0.333^***^	0.371^***^	0.562^***^	0.197^***^	0.511^***^	0.512^***^	1

Note:

* , *P* <  0.05;

** , *P* <  0.01;

*** , *P* <  0.0001.

### The cross‐lag model

**Fig 1:**
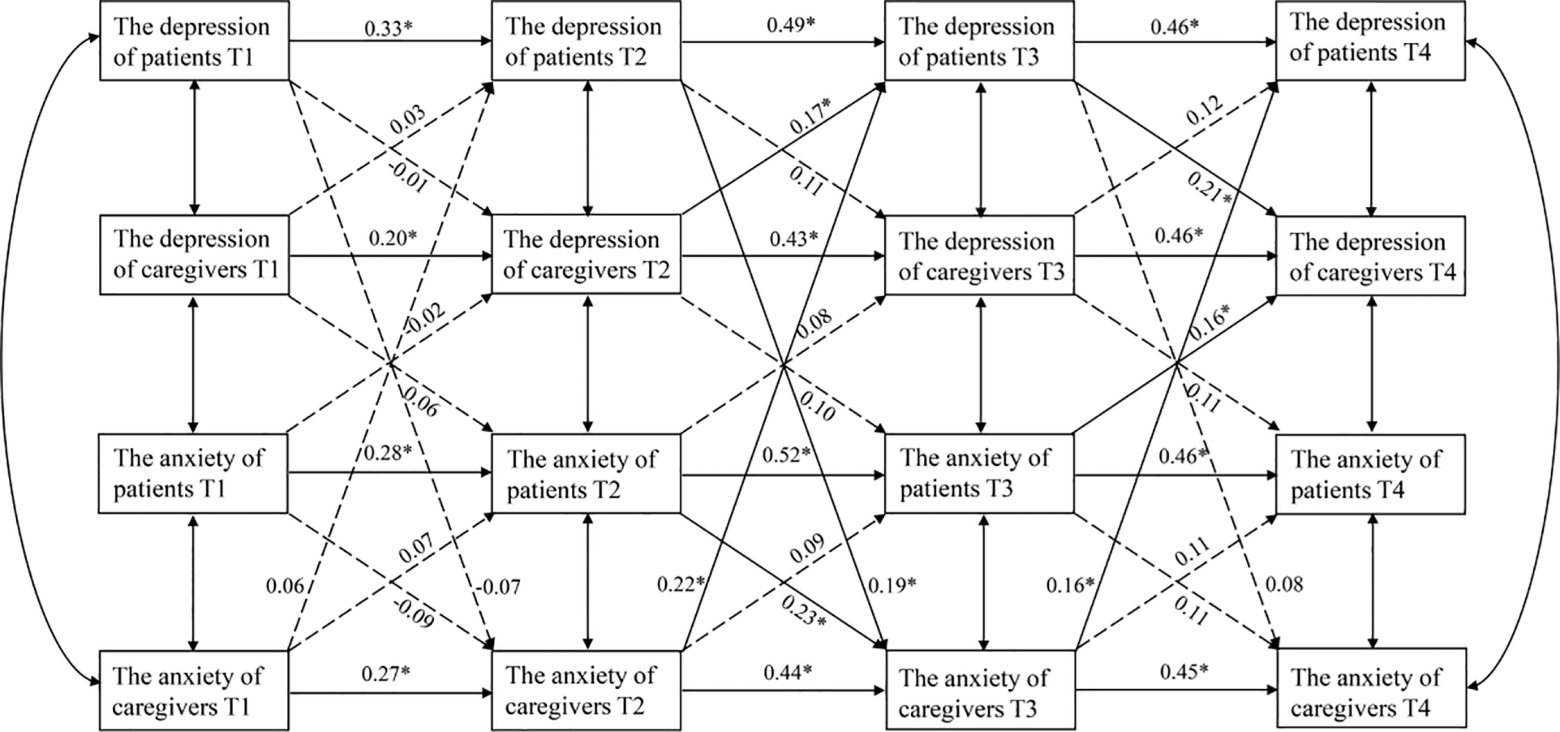
Cross-lag model of anxiety and depression between patients with colorectal cancer undergoing chemotherapy and family caregivers.

The cross-lagged model of depression and anxiety between colorectal cancer chemotherapy patients and their family caregivers is shown in Fig 1. The cross-lagged model fit results were as follows: χ^2^/df =  2.17, *P* =  0.014, GFI =  0.934, CFI =  0.968, RMSEA =  0.618, indicating a good fit. The paths in the model with significant positive predictive effects for cross-lagged effects were T2 patient anxiety to T3 caregiver anxiety (*β* =  0.23, *P* =  0.03); T3 patient anxiety to T4 caregiver depression (*β* =  0.16, *P* =  0.02); T2 patient depression to T3 caregiver anxiety (*β* =  0.19, *P* =  0.01); T3 patient depression to T4 caregiver depression (*β* =  0.21, *P* =  0.04); T2 caregiver anxiety to T3 patient depression (*β* =  0.22, *P* =  0.03); T3 caregiver anxiety to T4 patient depression (*β* =  0.16, *P* =  0.01); and T2 caregiver depression to T3 patient depression (*β* =  0.17, *P* =  0.01).

## Discussion

This study collected four-wave data to investigate the prospective and reciprocal relationships between anxiety and depression in colorectal cancer patients undergoing chemotherapy and their family caregivers. We found a high prevalence of anxiety and depression among both patients and family caregivers at the four follow-up time points. Furthermore, the anxiety and depression symptoms in both groups were found to be interrelated throughout the follow-up process. The study further emphasized the bidirectional relationships in mental health between chemotherapy patients and their caregivers.

This study found that colorectal cancer patients undergoing chemotherapy and their family caregivers exhibited high prevalence rates of depression and anxiety across the four-wave survey, generally higher than the results from previous meta-analyses on depression and anxiety prevalence among cancer patients [16] and caregivers [17]. Additionally, we observed that, over time, the levels of depression and anxiety in both patients and caregivers showed an overall increasing trend, which is consistent with previous research findings [18]. This phenomenon may be attributed to the chemotherapy context of this study, as the side effects of chemotherapy are a significant factor contributing to depression and anxiety in colorectal cancer patients [19]. As chemotherapy progresses, patients may experience more severe side effects, and the caregiving burden on family caregivers may also intensify, leading to further deterioration in mental health [20]. Additionally, stressful life events might be a significant cause of this phenomenon. Most of the patients and caregivers in this study were working individuals or those aged 30-60. These individuals may have had to shift their social roles or take on caregiving responsibilities due to the illness, conflicting with their original work and life arrangements [21]. Moreover, the significant economic burden of chemotherapy diminishes the earning capacity of patients and their caregivers, exacerbates financial conflicts, and disrupts family function and structure, which can lead to adverse emotional effects among family members [22]. As chemotherapy continues, these conflicts become more pronounced, resulting in the high prevalence rates of depression and anxiety and the increasing trend observed in this study.

Notably, during the T3-T4 phases, the anxiety scores of patients and the depression and anxiety scores of caregivers decreased. This suggests that the adverse emotions experienced by patients and their family caregivers during chemotherapy are not stable. This pattern is similar to the fluctuations observed in depression and anxiety among stroke patients and their caregivers [23]. This could be attributed to the fact that, after undergoing chemotherapy, patients and caregivers may not yet reaching the critical threshold of disease and caregiving burden and gradually adapted to the chemotherapy and caregiving routine, leading to a stabilization or slight reduction in depressive and anxious symptoms [24].

The results of this study also indicate that from the second wave onwards, the anxiety and depression of patients and their family caregivers begin to mutually influence each other, with several paths showing positive predictive effects. Previous reviews have also suggested that the negative emotions of cancer patients and their caregivers are bidirectionally influential [25]. A possible reason is that colorectal cancer patients undergoing chemotherapy and their family caregivers coexist within the same family system, forming an inseparable dyad that faces the disease together [26]. During chemotherapy, patients are in more complex situations and increasingly rely on their caregivers for emotional, physical, and financial support, which demands higher levels of caregiving knowledge and skills [27]. Consequently, the depression and anxiety of patients can affect their caregivers, leading to poorer mental health among caregivers.

Furthermore, the demands of patients are not unlimited; when they perceive their caregivers are burdened, they may hide their actual needs and avoid sharing cancer-related thoughts and feelings, a phenomenon known as “protective buffering” [28]. Therefore, when caregivers exhibit anxiety and depression that patients can detect, colorectal cancer patients may withdraw and reduce emotional expression, thereby exacerbating their anxiety and depression.

Our results also uncovered that the anxiety and depression of patients and caregivers in the first wave did not have a bidirectional impact on subsequent outcomes, which may be due to differences in measurement timing. This second wave of data was collected one month after the first chemotherapy session, which might have been a relatively short interval. At this point, the connection between colorectal cancer patients undergoing chemotherapy and their family caregivers may not yet have been strong enough to exhibit a considerable mutual influence [29].

### Limitations

This study has several limitations. First, as a single-center study, the generalizability of the findings may be limited to the specific population under investigation. Second, the study did not explore the longitudinal relationship between other negative emotions experienced by colorectal cancer patients undergoing chemotherapy and their family caregivers. Third, data on the levels of depression and anxiety in patients and their family caregivers prior to the initiation of chemotherapy were not collected, limiting the ability to assess pre-treatment emotional states. Lastly, an extended follow-up period could provide further insights into the evolving interrelationship of negative emotions between colorectal cancer patients and their family caregivers.

### Clinical implications

Based on the limitations of this study, we recommend that future related research adopt a more scientifically rigorous design, including multi-center and long-term follow-up studies, to further understand the psychological dynamics of colorectal cancer patients and their family caregivers. Additionally, baseline assessment data should be incorporated to enhance the persuasiveness of the research findings.

This study elucidated the longitudinal bidirectional relationship between anxiety and depression in colorectal cancer patients undergoing chemotherapy and their family caregivers. Future clinical interventions should consider both patients and caregivers as a single unit. Previous research has shown that dyadic psychological interventions are more effective than targeting only one individual [30]. In recent years, the forms of dyadic psychological interventions have been expanding. Traditional face-to-face and telephone interventions [31] and innovative e-health network interventions [32] have all been shown to improve the psychological health of patients and their caregivers.

Furthermore, attention should be given to the temporal trajectory of negative emotions within dyads. Early screening and ongoing psychological assessments at each chemotherapy time point, especially one month after the start of chemotherapy, are crucial. Healthcare professionals and psychological counselors should provide social support and personalized intervention measures at different times to accommodate the dynamic changes in negative emotions.

## Conclusion

The results of this study indicate that negative emotions such as anxiety and depression in colorectal cancer patients undergoing chemotherapy and their family caregivers exhibit dynamic changes at different stages of chemotherapy and have a longitudinal positive bidirectional relationship. Healthcare professionals should scientifically assess the negative emotions of colorectal cancer patients and their family caregivers at different stages of chemotherapy, providing targeted intervention guidance and emphasizing their physical and mental health to improve treatment outcomes and quality of life for both patients and caregivers. Future research should adopt multi-center, large-sample, and extended follow-up study designs to explore the longitudinal bidirectional relationship of negative emotions in colorectal cancer patients undergoing chemotherapy and their family caregivers.
